# Speech apraxia and oral apraxia: association or dissociation? A multivariate lesion–symptom mapping study in acute stroke patients

**DOI:** 10.1007/s00221-021-06224-3

**Published:** 2021-10-15

**Authors:** Martina Conterno, Dorothee Kümmerer, Andrea Dressing, Volkmar Glauche, Horst Urbach, Cornelius Weiller, Michel Rijntjes

**Affiliations:** 1 Clinic of Neurology and Neurophysiology, Medical Centre-University of Freiburg, Faculty of Medicine, University of Freiburg, Breisacher Straße 64, 79106 Freiburg im Breisgau, Germany; 2 Freiburg Brain Imaging Centre, University of Freiburg, 79106 Freiburg im Breisgau, Germany; 3 BrainLinks-BrainTools Cluster of Excellence, University of Freiburg, 79110 Freiburg im Breisgau, Germany; 4 Department of Neuroradiology, Medical Centre-University of Freiburg, Faculty of Medicine, University of Freiburg, Freiburg im Breisgau, Germany

**Keywords:** Speech apraxia, Oral apraxia, Stroke, Support vector regression, Multivariate lesion–symptom mapping, Insula, Precentral gyrus

## Abstract

The anatomical relationship between speech apraxia (SA) and oral apraxia (OA) is still unclear. To shed light on this matter we studied 137 patients with acute ischaemic left-hemisphere stroke and performed support vector regression-based, multivariate lesion–symptom mapping. Thirty-three patients presented with either SA or OA. These two symptoms mostly co-occurred (*n* = 28), except for few patients with isolated SA (*n* = 2) or OA (*n* = 3). All patient with either SA or OA presented with aphasia (*p* < 0.001) and these symptoms were highly associated with apraxia (*p* < 0.001). Co-occurring SA and OA were predominantly associated with insular lesions, while the insula was completely spared in the five patients with isolated SA or OA. Isolated SA occurred in case of frontal lesions (prefrontal gyrus and superior longitudinal fasciculus), while isolated OA occurred in case of either temporoparietal or striatocapsular lesions. Our study supports the notion of a predominant, but not exclusive, role of the insula in verbal and non-verbal oral praxis, and indicates that frontal regions may contribute exclusively to verbal oral praxis, while temporoparietal and striatocapsular regions contribute to non-verbal oral praxis. However, since tests for SA and OA so far intrinsically also investigate aphasia and apraxia, refined tests are warranted.

## Introduction

The anatomical and linguistic basis of motor planning for the oral system is still a matter of discussion. The disruption of motor planning pathways that lead to an impairment of oral praxis can manifest itself as either apraxia of the speech-related oral movements (speech apraxia) or as apraxia of the non-speech-related oral movements (oral apraxia), or both. The anatomical relationship between these two syndromes has not yet been fully clarified and is the focus of this study.

Speech apraxia (SA), or apraxia of speech, is a motor speech disorder defined as a deficit in planning and programming sensory–motor movements for speech (Darley 1982; Duffy 2005). Diagnosing SA is challenging due to its frequent co-occurrence with aphasia (a higher-level linguistic impairment) and dysarthria (an impairment in speech execution and control). In SA, aphasia and dysarthria speech production is impaired in different ways, but speech sound errors that occur in all three disorders can be present similarly. The most relevant features of SA that have gained broad consensus for differential diagnosis include reduced overall speech rate, phoneme distortions and distorted substitutions, additions or complications, syllable segregation with extended intra- and intersegmental durations, and equal stress across adjacent syllables; these features typically increase with greater syllable length and motor complexity (Basilakos 2018; Ziegler et al. 2020). The most common causes of SA are vascular lesions such as stroke; less frequently, this symptom may also result from tumours, trauma or neurodegenerative diseases (Dronkers 1996; Josephs et al. 2012).

At an anatomical level, the region responsible for SA was first described in the left superior precentral gyrus of the insula in a lesion overlap study in stroke patients (Dronkers 1996). Patients with severe SA were found to have larger lesions additionally involving Broca’s area, the basal ganglia, the external and internal capsule, and the superior longitudinal fasciculus (SLF) (Ogar et al. 2006). It has been objected that, since SA typically co-occurs with aphasia (De Renzi et al. 1966; Dronkers 1996), insular involvement reflects the concomitance of aphasia (Uddin et al. 2017) rather than being causally related to SA. Accordingly, in a lesion overlap study on chronic SA patients without aphasia, the left premotor and motor cortex, and not the insula, were the areas of maximal overlap (Graff-Radford et al. 2014).

However, the lesion overlap approach has several limitations. First, since SA (in particular chronic SA) is usually caused by a large stroke (Pedersen et al. 2001; Ogar et al. 2006; Trupe et al. 2013), the area of greatest overlap might just reflect the fact that the insula is one of the most commonly injured areas in large middle cerebral artery (MCA) strokes. Second, lesion overlap studies only consider the probability of a lesion in patients with a particular symptom, and not the probability of the symptom in case of a particular lesion (Hillis et al. 2004b; Kodumuri et al. 2016). For this reason, Dronkers (1996) added an overlap of the lesions of patients without SA to her analysis, as control.

Trying to overcome these limitations, several studies, based on either acute or chronic stroke patients with or without aphasia, analysed lesion location of SA using statistically more powerful approaches, such as Chi-squared test, voxel-based lesion-–symptom mapping (VLSM) or cerebral blood volume-based regression approaches. As a result, SA was associated either with the left inferior frontal gyrus (IFG) (Hillis et al. 2004b; Richardson et al. 2012; Trupe et al. 2013), the precentral gyrus (Basilakos et al. 2015; Itabashi et al. 2016) or, in a recent study, again the insula and the dorsal arcuate fasciculus (Chenausky et al. 2020). Additional evidence has been conveyed by studies on neurodegenerative, progressive SA (Josephs et al. 2006, 2012), which demonstrated grey matter atrophy predominantly in the dorsal lateral premotor cortex (PMC) and in the supplemental motor area (SMA).

Findings from functional imaging studies demonstrated that motor speech programming tasks can elicit activation of the insula and the IFG (Bonilha et al. 2006; Ackermann and Riecker 2010), while New et al. (2015) reported that stroke patients with SA have reduced resting state connectivity between the left and right ventral premotor regions, and that reduced PMC functional connectivity is related to SA severity.

Ultimately, there is still no consensus on which brain areas are responsible for causing speech apraxia, and the roles of the insula, the IFG and the PMC are yet to be clarified.

Oral apraxia (OA), or buccofacial apraxia, is the inability to imitate or perform volitional non-verbal oromotor movements (e.g. coughing, blowing, clicking the tongue) on command, due to a deficit in motor planning and sequencing, despite intact vegetative and sensory–motor function of the oral tract and preserved task comprehension. Patients with OA attempt to respond to the requested tasks but fail in various ways (e.g. effortful groping, inconsistent trial and error attempts, off-target response or speaking instead of performing the task) (Biniek et al. 1992; Bizzozero et al. 2000; Whiteside et al. 2015).

As for SA, OA has been mostly studied in stroke patients, but reports are fewer. Older CT studies described an association with diffuse frontotemporal regions, the insula and subcortical regions, such as the basal ganglia, the internal capsule and the anterior periventricular white matter, prevalently in the left hemisphere (Tognola and Vignolo 1980; Raade et al. 1991; Alexander et al. 1992; Maeshima et al. 1997; Bizzozero et al. 2000). One case report of isolated OA showed a lesion in the ventral portion of the left PMC (Kwon et al. 2013). Regarding the occurrence of lesions in the right hemisphere (Bizzozero et al. 2000), a partial bilateral involvement of these regions in regulating non-verbal oral praxis has been described (Wildgruber et al. 1996; Bonilha et al. 2006). In summary, similar to SA, the precise localization of lesions responsible for OA remains unclear and some regions are implicated in both syndromes.

Nearly all studies consider oral praxis exclusively in relation to speech and language deficits, while the clinical and anatomical relationship between SA, OA and limb apraxia has been less documented. Previous studies reported for OA frequencies of co-occurring limb apraxia around 60% (Raade et al. 1991), and for SA around 50% in stroke patients (Dronkers 1996) and 30% in primary progressive SA (Josephs et al. 2012). Older studies reported similarities between lesions causing OA and speech planning areas rather than limb praxis areas (Raade et al. 1991; Alexander et al. 1992). The already cited study by Botha found an association of limb apraxia and OA among neurodegenerative patients without SA, which could indicate the disruption of similar mechanisms in non-verbal oral praxis and limb praxis (Botha et al. 2014).

To better understand the processes underlying disruption of verbal and non-verbal oral praxis, it is crucial to address the question whether SA and OA belong to the same anatomical unit or not. Reportedly, SA and OA often co-occur (McNeil et al. 1990; Dronkers 1996; Whiteside et al. 2015). The current literature on the relationship between SA and OA has reached diverging conclusions (Ballard et al. 2000, 2003; Ziegler 2003; Ziegler et al. 2012; Whiteside et al. 2015), leading to two alternative theories: a task-dependent model, which supports the existence of separate sensory–motor systems for verbal and non-verbal oral movements, and a task-independent, integrative model, which proposes a universal and overlapping sensory–motor system responsible for all functions of the speech apparatus, irrespective of their purpose. On one side, supporting the task-dependent hypothesis, dissociations between SA and OA have been described at a clinical level in both stroke and neurodegenerative patients (Botha et al. 2014; Galluzzi et al. 2015). The co-occurrence of SA and OA might be merely due to the proximity of the areas responsible for planning these two different types of oral movements (Ziegler 2003). Whiteside and colleagues (2015) reinforced this hypothesis by observing, basing on an extended battery of verbal and non-verbal tasks, double dissociations between SA and OA and only a moderate positive association between the severity of these symptoms. On the other side, supporters of the task-independent, integrative model point out that dissociations between SA and OA might depend on the fact that the current clinical tests for OA require a lower degree of movement difficulty, compared to speech testing, and have therefore lower sensitivity (Ballard et al. 2000, 2003). Evidence from a resting state fMRI study (New et al. 2015) supports the idea that neural networks behind speech and non-speech praxis (left PMC—right anterior insula) overlap to some degree.

Other than these indirect or behavioural data on the relationship between SA and OA, the question if SA and OA are anatomically associated or dissociated has not been yet sufficiently investigated through direct comparisons of acute lesions in large MRI-based studies. The matter has been addressed by Botha in neurodegenerative disease (Botha et al. 2014), supporting the hypothesis of dissociation between verbal and non-verbal oral praxis: OA was associated with bilateral atrophy of the prefrontal cortex, while previous data from the same group had shown an association of neurodegenerative SA with more posterior frontal regions, namely the superior lateral PMC and the SMA (Josephs et al. 2012). On the contrary, a MRI/CT study on mixed subacute and chronic, mainly ischaemic-related brain lesions obtained a prominent involvement of the insula in both SA and OA, supporting the idea of a common anatomical substrate (Yadegari et al. 2014). It has to be pointed out that the study by Botha was based on neurodegenerative patients, whose brain damage is based on different mechanisms from stroke, while the study by Yadegari examined chronic lesions, which hampers the evaluation of a direct lesion–symptom relationship, due to clinical recovery and neuroanatomical reorganization (Hillis et al. 2006; Martin et al. 2017). To draw a direct link between lesion and symptom, it is crucial to examine acute, isolated and circumscribed lesions.

The aim of our study was therefore to investigate the anatomical relationship between SA and OA, based on a large sample of patients with acute left hemisphere ischaemic stroke lesions.

## Materials and methods

### Patients

Patients with ischaemic, thromboembolic stroke were consecutively recruited between February 2011 and April 2015 from the Stroke Unit of the Department of Neurology and Neurophysiology at the University Medical Centre of Freiburg, Germany. We focused on the left hemisphere, since SA and OA predominantly occur after left-sided brain lesions (Tognola and Vignolo 1980; Dronkers 1996; Ogar et al. 2005).

Overall, 683 patients with acute first-ever middle cerebral artery (MCA) ischaemic stroke in the left hemisphere were screened. Patients with previous or bilateral infarcts, or pre-existing speech/language pathologies were not considered. From this cohort, 546 patients were excluded, based on the following exclusion criteria: (1) age > 90 years (*n* = 16); (2) inability to perform clinical testing owing to reduced general health status (intubation, hemicraniectomy, etc., *n* = 89); (3) previous invalidating neurological pathologies (i.e. normal pressure hydrocephalus, hereditary diseases, multiple sclerosis, movement disorders, severe microangiopathy, traumatic brain injury, cerebral bleeding, etc.) (*n* = 154); (4) mother tongue other than German (*n* = 21); (5) major cognitive impairments or severe hearing/visual deficits (*n* = 42); (6) practical reasons (contraindications for MRI, declined participation, lack of cooperation due to psychiatric diseases or delirium) (*n* = 195); (7) co-occurring infratentorial infarct (*n* = 5); (8) incomplete evaluation of SA or OA, or no speech production due to severe global aphasia (*n* = 24).

The resulting 137 patients had all undergone speech, language and limb apraxia testing during their hospital stay (speech/language testing: mean symptom-test delay: 4.4 days, standard deviation—SD—2.3, min/max 0–10 days after stroke; apraxia testing: mean symptom-test delay: 4.9 days, SD 2.0, min/max 1–11 days after stroke). Data regarding age, gender and stroke severity (National Institutes of Health Stroke Scale, NIHSS) were collected.

Speech and language impairments were tested by experienced speech therapists from our neurological clinic, specialized in adult neurological disorders. The results of their testing were checked for reliability and plausibility by D.K.

Aphasia was diagnosed using the Aachen Aphasia Test (AAT) for the German language (Huber et al. 1984) or the Aachen Aphasia Bedside Test for more severely affected patients (Biniek et al. 1992). Dysarthria was diagnosed on the basis of the Frenchay Dysarthria Assessment (Enderby 1980) adapted for the German language. SA was diagnosed by an assessment of apraxia of speech for the German language (Lauer and Birner-Janusch 2007) with an evaluation of articulation, prosody, diadochokinesia, speech behaviour, respiration, and phonation of the spontaneous speech, reading of a text and repetition of words with increasing complexity regarding word length, complexity of syllables and lexicality. Errors were classified according to the following criteria: articulation (consonant and vowel distortions, substitutions, additions, omissions, perseverative substitutions, anticipatory substitutions, sound prolongations, inconsistency of articulation errors), rate and prosody (slow overall rate, errors of stress assignment, altered stress), fluency (initiation problems, syllable segmentation, lengthened intersegment duration), speech behaviour (visual or audible groping, effortful speech, self-correction, other non-related compensatory movements) and intelligibility. OA was diagnosed on the basis of the subtest MuMo of the Aachen Aphasia Bedside Test (Biniek et al. 1992), testing for buccofacial apraxia comprising prompts for oral movements. Patients were asked to perform non-verbal tasks on command on a ten-item measure such as blowing, smiling, or licking the lips. Tests for both SA and OA were performed primarily on verbal command and, if the patient showed difficulties understanding the prompts, to overcome the impact of speech comprehension deficits, through imitation.

For the purposes of the present study, since not all subtests used to diagnose SA or OA provide continuous data, speech and language impairments were considered as dichotomous values (presence/absence of aphasia, dysarthria, SA or OA). As a further characterization of aphasia, in particular to clarify the role of the impairment of semantic and comprehension skills in our sample, we additionally selected as output variable “speech comprehension” from the AAT. This variable was again considered as dichotomous value (impaired/not impaired, based on the Stanine scores provided by the AAT). Speech comprehension could not be assessed in ten of the patients because of insufficient cooperation or too severe clinical conditions (only the Token Test was completed).

Limb apraxia was tested by experienced occupational therapists. Since a composite “apraxia score” analogous to aphasia from the AAT is not established, limb apraxia was assessed as a deficit of either “imitation of meaningless postures” or “pantomime of tool use”. The patients were tested for imitation of meaningless hand and finger postures and pantomime of tool use (Goldenberg and Strauss 2002; Goldenberg and Karnath 2006; Bartolo et al. 2008) using the modified apraxia tests developed by Hoeren et al. (2014). Imitation of meaningless postures and pantomime of tool use were considered as dichotomous values (impaired/not impaired), based on the cutoffs established in the above-mentioned study (Hoeren et al. 2014). Two patients could not be tested for either imitation or pantomime because of insufficient cooperation or too severe clinical conditions. In testing for pantomime, nine patients were additionally excluded because of impaired object recognition, i.e. < 27 points in the subtest 11 of the Birmingham Object Recognition Battery (BORB-11, Riddoch and Humphreys 1993), analogously to the approach adopted in previous studies from our laboratory (Martin et al. 2017; Dressing et al. 2018).

### Statistics

To describe our sample, we used mean and standard deviation (SD) for continuous and discrete variables, absolute frequency and percentage for categorical variables. To compare subgroups, we used the Chi-square test for binomial variables, or the Mann–Whitney *U* test for continuous/categorical variables. Statistical analyses were performed using the IBM Statistical Package for Social Sciences (SPSS, version 24.0, Armonk, NY).

### Imaging

MRI scans were performed within a mean of 2.5 days after stroke onset (SD 2.8, min/max 0–9 days after stroke) on either a 3 T Trio scanner or a 1.5 T Avanto scanner (Siemens, Germany). To delineate lesions, diffusion-weighted imaging (DWI) sequences (23 slices, matrix = 128 × 128 pixel, voxel size = 1.8 × 1.8 × 5 mm, repetition time = 3.1 s, echo time = 79 ms, flip angle = 90, six diffusion-encoding gradient directions with a b-factor of 1000 s/mm^2^) were acquired. We also acquired fluid-attenuated inversion recovery (FLAIR) images (repetition time = 9000 ms, echo time = 93.0 ms, flip angle = 140°, matrix 200 × 256 pixel, voxel size = 0.94 × 0.94 × 5.00 mm, 23 slices) and, as a requirement for spatial normalization, a high-resolution T1 anatomical scan (MPRAGE, repetition time = 2200 ms, echo time = 2.15 ms, flip angle = 12°, matrix = 256 × 256 pixel, voxel size = 1 × 1 × 1 mm, 176 slices).

Lesion analysis was performed using the same approach described in previous studies from our laboratory (Hoeren et al. 2014; Beume et al. 2017). Firstly, the DWI lesions were drawn using a customized region-of-interest toolbox in SPM8 (http://www.fil.ion.ucl.ac.uk/spm/software/spm8). Customized intensity thresholds were applied to have an optimal match between binary lesion map and diffusion-restricted brain lesion. Lesion maps were then compared directly to patients' native scans (DWI sequences) in MRIcron (http://people.cas.sc.edu/rorden/mricron/index.html) and adjusted if needed. To make the lesions spatially comparable with each other we normalized the DWI sequences to anatomical high-resolution T1 scans, which were segmented using the VBM8 toolbox (r435; http://dbm.neuro.uni-jena.de/vbm8/). Using the DARTEL (diffeomorphic anatomical registration through exponentiated lie algebra) approach (Ashburner 2007) implemented in VBM8, deformation field parameters for nonlinear normalization were then computed into the stereotactic MNI (Montreal Neurological Institute) standard space. If the T1 scans had insufficient quality, normalization parameters were instead acquired from FLAIR sequences. After normalization, the lesion maps were again visually inspected to make sure their localization matched patients' native scans, and were adjusted if needed.

We then performed a support vector regression-based, multivariate lesion–symptom mapping analysis (SVR-LSM), using a toolbox recently developed by De Marco and Turkeltaub (DeMarco and Turkeltaub 2018) and based on Zhang’s approach (Zhang et al. 2014). This approach, in comparison to the traditional univariate voxel-based lesion–symptom mapping (VLSM) analysis, has shown higher sensitivity for identifying lesion–behaviour relations, accounting for intervoxel correlations and allowing to identify multivariate interactions between lesion location and studied symptom (Zhang et al. 2014). The toolbox creates a voxelwise map of raw regression *β* values and performs statistical thresholding to obtain significance thresholds for single voxels and cluster extents. The resulting *β* values were thresholded at *p* < 0.005 and corrected for cluster size at *p* < 0.05, both based on 3000 permutations. The analysis was restricted to voxels with at least ten overlapping lesions. Lesion volume correction was included by regressing lesion volume out of both, behavioural data and lesion maps. This correction is particularly relevant in our study, since SA and OA following stroke tend to be associated with larger lesions (Pedersen et al. 2001; Ogar et al. 2006; Trupe et al. 2013).

Results are displayed on an in-house average template of 50 nonlinearly normalized T1 scans from a sample of healthy subjects who had participated in various other studies in our laboratory (mean age = 47 ± 20.75 years, range = 22–84 years, 25 male) (Hoeren et al. 2014; Beume et al. 2017). To estimate the location of the different brain regions we used the anatomical templates provided by MRIcron.

## Results

### Clinical data

Clinical data of the sample are shown in Table 1. SA and OA occurred in 22 and 23% of the patients, respectively. Aphasia was present in almost half of the patients (47%), as well as an impairment of speech comprehension (42%). Imitation was impaired in 44% of the sample, but pantomime only in 28%.

**Table 1:** Demographic and clinical data

Demographic and clinical data (*n* = 137)	Mean (SD)	Range	Absolute frequency (%)
Age	64.6 (14.5)	21.9–85.7	
Gender (male)			89 (65)
NIHSS at admission	6.8 (5.6)	0–24	
NIHSS at discharge	2.7 (2.8)	0–15	
Lesion volume (cc)	25.0 (34.4)	0.3–244.9	
SA			30 (22)
OA			31 (23)
Aphasia			65 (47)
Impaired comprehension			53 (42^1^)
Apraxia			
Impaired pantomime			35 (28^2^)
Impaired imitation			60 (44^3^)
Dysarthria			49 (36)

*NIHSS* National Institutes of Health Stroke Scale, *SD* standard deviation

^1^*n* = 127; ^2^*n* = 126; ^3^*n* = 135 (see "Patients")

Thirty-three patients presented with either SA or OA, and these two symptoms mostly co-occurred. In three patients, the initial finding was isolated SA (i.e. without OA), but this could not be confirmed after a plausibility check (by D.K.). These patients were re-evaluated and it was concluded that all three presented with both SA and OA. The discrepancies related to the judgement whether there was mild oral apraxia or none at all. For all other patients, the diagnosis of speech therapists concurred. Results of the interrater reliability using Cohen’s *κ* yielded high agreement on the presence of SA (*κ* 1.000) and the presence of OA (*κ* 0.935).

All patients with either SA or OA had co-occurring aphasia, and approximately 70% of them had an impairment of pantomime or imitation (72% and 73%, respectively). We divided these patients (*n* = 33) in three subgroups, which we analysed separately (Table 2): patients showing both deficits (“SA + OA”, *n* = 28), patients with SA without OA (isolated SA, iSA, *n* = 2) and patients with OA without SA (isolated OA, iOA, *n* = 3).

**Table 2:** Characteristics of patients with SA and/or OA

	SA + OA (*n* = 28)			iSA pt. 1	iSA pt. 2	iOA pt. 1	iOA pt. 2	iOA pt. 3
	Mean (SD)	Range	%					
Age	66.8 (11.9) (n.s.)	32.8–85.7		77.6	35.1	64.4	59.1	81.8
Gender (male)			71 (n.s.)	+	–	+	+	+
NIHSS on admission	11.1 (5.9)*	3–24		4	12	3	15	22
NIHSS on discharge	5.0 (3.5) *	1–15		2	6	3	3	9
Lesion volume (cc)	66.9 (51.1)*	5.9–244.9		27.3	45.5	55.6	3.4	13.5
Aphasia			100*	+	+	+	+	+
Impaired comprehension			96^1^*	+	+	+	n.t.	+
Impaired pantomime			81^2^*	–	–	n.t.	–	+
Impaired imitation			75*	+	–	+	–	+
Dysarthria			32 (n.s.)	–	+	–	–	+

The SA + OA group (*n* = 28) was compared with the rest of the sample using either the *χ*^2^ test or the Mann–Whitney *U* test (* = *p* < 0.001). For the iSA/iOA patients the absolute values are shown (+ = presence of the symptom, – = absence of the symptom)

*N.t.* not tested, *n.s.* not significant, *pt.* patient, *SD* standard deviation

^1^*n* = 23; ^2^*n* = 21 (see "Patients")

#### SA + OA

We compared the SA + OA group with the rest of our sample using either the chi-square test or the Mann–Whitney *U* test (Table 2). The SA + OA group did not differ from the rest of the sample for age or gender. Patients with SA + OA were more severely neurologically impaired (higher NIHSS) than the rest of the sample, both on admission (*p* < 0.001) and on discharge (*p* < 0.001), and had larger lesions (*p* < 0.001).

The occurrence of limb apraxia (both pantomime and imitation) and aphasia was significantly higher in the *SA* + *OA* group (*p* < 0.001) than in the rest of the sample, while no significant association was found between SA + OA and dysarthria.

#### iSA and iOA

Because of the small size of the iSA and iOA groups (2 and 3 patients, respectively), the features of each single patient are shown (Table 2). Four of the five patients with either iSA or iOA manifested an impairment of speech comprehension, one could not be tested because of too severe clinical impairment (see "Patients"). In the two patients with *iSA*, pantomime was not impaired and only one patient had an impairment of imitation. In the three patients with iOA, one had an impairment of pantomime and two had an impairment of imitation (one patient could not be tested for pantomime since BORB-11 score < 27, see "Patients").

### Lesion analysis

Attempting to differentiate the anatomy of SA and OA, we considered the above described subgroups separately: SA + OA (*n* = 28), iSA (*n* = 2) and iOA (*n* = 3).

#### SA + OA

**Fig. 1:**
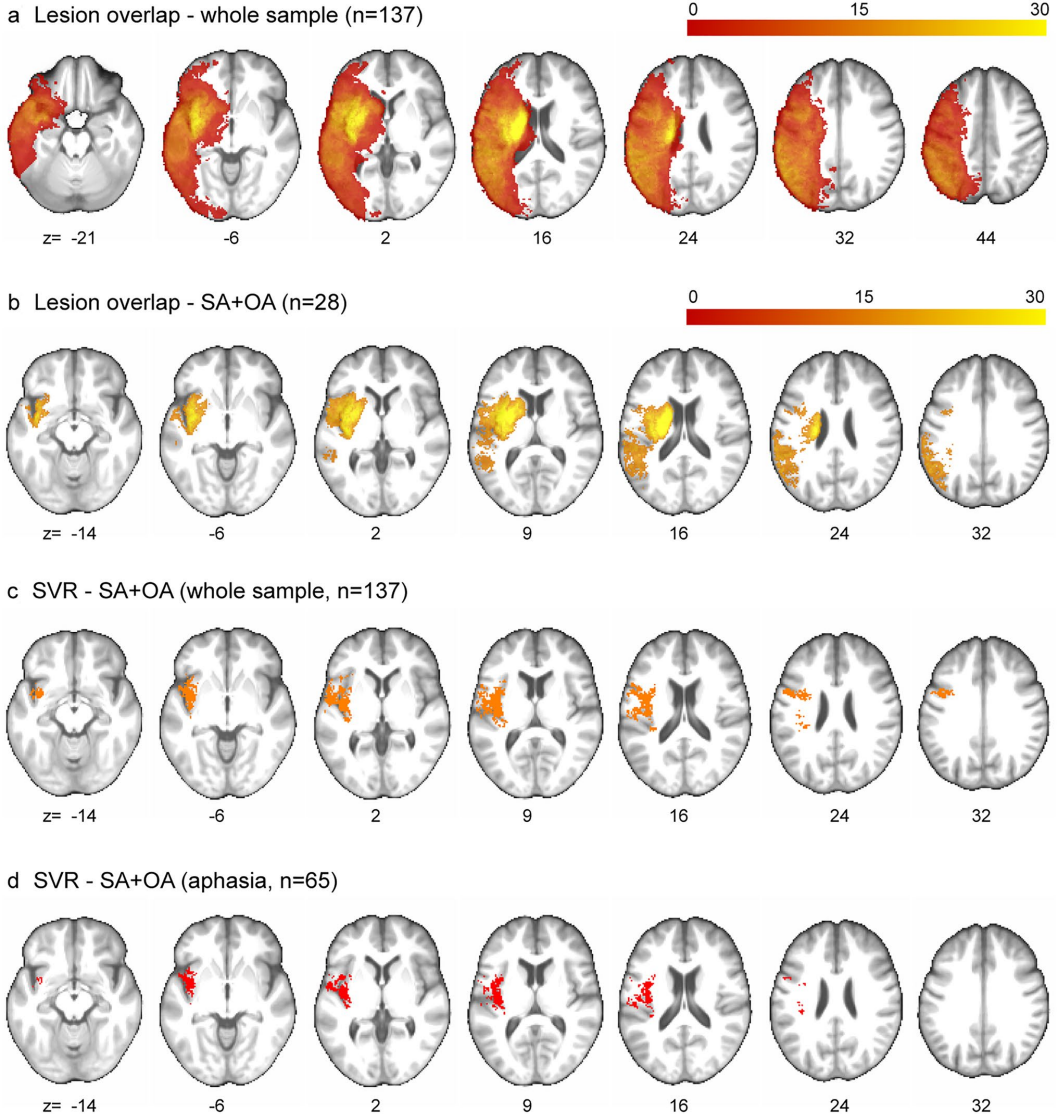
Lesion maps and analysis for the SA + OA group. For each slice the MNI coordinate (*z*) is given. **a** Lesion overlap of the whole patient sample. The colour bar shows the colour range corresponding to the number of overlapping lesions. **b** Lesion overlap of the patients with SA + OA (*n* = 28). The colour bar shows the colour range corresponding to the number of overlapping lesions. **c** SVR-LSM clusterwise analysis for SA + OA in comparison to the whole sample (*n* = 137). Six patients were excluded for having no voxels inside the minimum lesion cut-off mask. The resulting SVR-*β* values were thresholded at *p* < 0.005 and corrected for cluster size at *p* < 0.05, both based on 3000 permutations. **d** SVR-LSM clusterwise analysis for SA + OA within the group of aphasic patients (*n* = 65), i.e. comparison between the lesions of aphasic patients with SA + OA and the lesions of aphasic patients without SA + OA. One patient was excluded for having no voxels inside the minimum lesion cut-off mask. The resulting SVR-β values were thresholded at *p* < 0.005 and corrected for cluster size at *p* < 0.05, both based on 3000 permutations

The lesion overlap maps for the whole sample and for the SA + OA group are shown in Fig. 1a, b.

The results of the SVR-LSM clusterwise analysis for the SA + OA group compared to the rest of the sample (*n* = 137) are shown in Fig. 1c. SA + OA were significantly associated with a cluster including a large portion of the insula and parts of the precentral gyrus (Brodmann area, BA, 6) at its border with the IFG (BA 44), of the Rolandic operculum and of white matter fibres (external/extreme capsule, SLF). Some significant voxels were additionally found in the superior temporal gyrus (STG), the internal capsule, the corona radiata and the transverse temporal gyri.

Since all patients with either SA or OA had co-occurring aphasia, it remained problematic to differentiate the contribution of this highly associated symptom to the SVR-LSM results. To reduce the effect of co-occurring aphasia, we performed again the same SVR-LSM analysis for SA + OA, testing it this time only within the group of patients presenting with aphasia (*n* = 65)—namely, we compared the lesions of aphasic patients with SA + OA to the ones of aphasic patients without SA + OA (Fig. 1d). As a result, the insula remained the region that was most extensively associated with SA + OA. In comparison to the whole population analysis, only very few additional voxels remained associated with SA + OA within the aphasia group, covering the precentral gyrus/IFG, the rolandic operculum, the external/extreme capsule and the corona radiata. No significant voxels were found in the STG or the transverse temporal gyri.

#### iSA and iOA

**Fig. 2:**
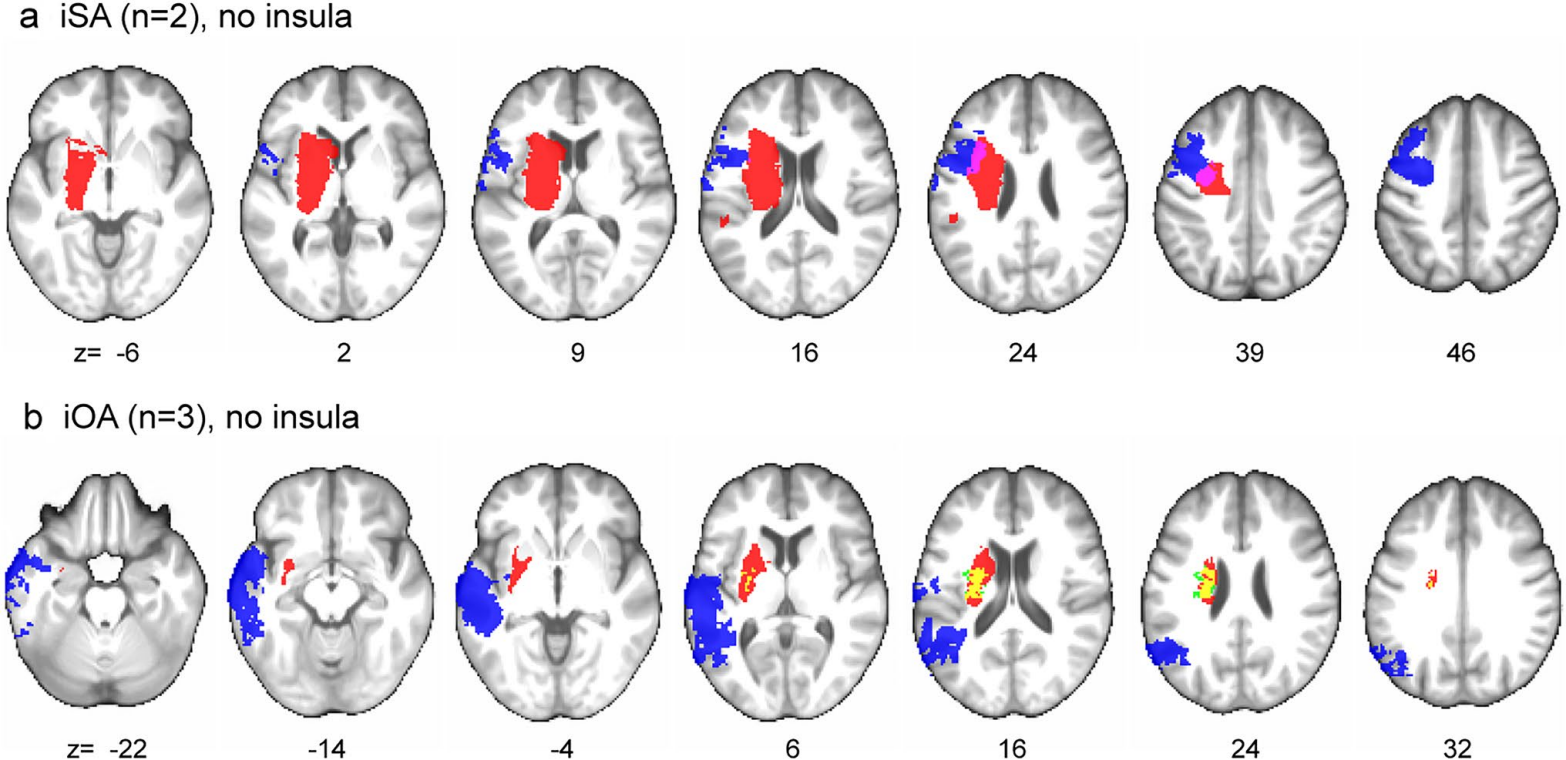
Lesion maps for iSA and iOA. For each slice the MNI coordinate (z) is given. **a** Lesion overlap for iSA (2 patients; patient 1 in blue, patient 2 in red, overlap region in pink). **b** Lesion overlap for iOA (3 patients; patient 1 in blue, patient 2 in green, patient 3 in red, overlap region between patient 2 and 3 in yellow)

For the subgroups of patients with iSA and iOA, because of the reduced sample size, overlap analyses were performed (Fig. 2). Interestingly, none of the five patients with isolated deficits showed an involvement of the insula. Patient 1 with iSA had a relatively circumscribed frontal lesion involving the precentral and middle frontal gyri, the IFG and the adjacent white matter (SLF); patient 2 with iSA had a mainly subcortical lesion including the basal ganglia, the internal capsule and the corticospinal tract, reaching into the frontal cortex. The two lesions overlapped in a small part of the precentral gyrus and in the adjacent white matter (SLF) (Fig. 2a). Patient 1 with iOA had a large temporoparietal lesion involving the superior, middle and inferior temporal gyri, including the anterior temporal lobe (ATL), and parts of the superior parietal lobule (SPL), the posterior inferior parietal lobule (IPL) and the inferior longitudinal fasciculus. Patient 2 with iOA had a small subcortical lesion in the basal ganglia (putamen, caudate nucleus) reaching into the internal and external/extreme capsules and the superior corona radiata. Patient 3 with iOA showed, similarly, a slightly bigger subcortical lesion involving again the basal ganglia (putamen, caudate nucleus and globus pallidus), the internal and external/extreme capsules and the corona radiata. The lesions of these last two patients overlapped almost completely, whereas there were no overlapping voxels between these two and the first temporoparietal lesion (Fig. 2b).

## Discussion

We focused on the anatomical relationship between SA and OA in a sample of 137 acute stroke patients. On one side, supporting the hypothesis that SA and OA belong to the same functional and anatomical unit, we observed a strong clinical association between these two symptoms (28 out of 33 patients), centred on the insula. On the other side, supporting the hypothesis that SA and OA are functionally and anatomically dissociated, we observed five patients with isolated symptoms (iSA or iOA), with lesions distributed over several areas, but excluding the insula.

In the SVR-LSM analysis, co-occurring SA and OA (SA + OA) were mostly associated with wide areas of the insula. Further associations were found with frontotemporal regions (i.e. precentral gyrus, opercular region, STG) and with adjacent white matter fibres, such as the external/extreme capsule and the SLF (Fig. 1c). The predominant association of SA with the insula is consistent with several studies on this symptom (Dronkers 1996; Ogar et al. 2006; Chenausky et al. 2020) and, in particular, our results included the superior precentral gyrus of the insula, in line with the first findings on the anatomy of SA (Dronkers 1996).

The involvement of the precentral gyrus in SA is also in line with recent evidence, identifying the left premotor cortex as the region responsible for SA in stroke (Graff-Radford et al. 2014; Basilakos et al. 2015; Itabashi et al. 2016) and in neurodegenerative disease (Josephs et al. 2006, 2012).

In OA the predominant involvement of the insula and the frontal region, including the precentral gyrus, is again in line with previous findings (Tognola and Vignolo 1980; Raade et al. 1991; Alexander et al. 1992; Maeshima et al. 1997; Ackermann and Riecker 2010; Kwon et al. 2013) and thus differs from the results obtained by Botha on neurodegenerative disease (Botha et al. 2014). This difference may be due to the fact that neurodegenerative disease is based on different pathogenetic processes from stroke.

However, all our subjects with either SA or OA had co-occurring aphasia, consistently with previous evidence (De Renzi et al. 1966; Tognola and Vignolo 1980; Dronkers 1996). It can be thus objected that our SVR-LSM results for SA + OA rather reflect the co-occurrence of aphasia, and not a direct association, in particular since insular involvement has been described in both speech and language processing (Kümmerer et al. 2013; Oh et al. 2014). After restricting our SVR-LSM analysis to the aphasic patients, with or without SA + OA, to reduce the effect of co-occurring aphasia, the insula remained the region to be predominantly associated with SA + OA. This result supports the hypothesis that this region actually plays a relevant role in verbal and non-verbal oral praxis (Fig. 1d).

Interestingly, none of the five patients with iSA or iOA had a lesion involving the insula (Fig. 2). Furthermore, in the SVR-LSM results for both the whole population and the aphasia subgroup analysis, SA + OA was associated with frontal regions and white matter fibres as well, indicating that the insula is not the only region involved in causing these symptoms. Thus, our results so far support the predominant, but not exclusive, role of the insula in both verbal and non-verbal oral praxis.

The lesions of the patients with iSA or iOA (5 out of 33), which did not show insular damage, bring further information on the regions that may be involved in verbal and non-verbal oral praxis. Lesions of the patients with iSA were located in the frontal lobe and the basal ganglia/internal capsule, overlapping in the precentral gyrus and in the adjacent white matter, while lesions of the patients with iOA were either temporoparietal or, again, subcortical (basal ganglia and adjacent white matter, including the internal and external/extreme capsules). Considering this information, it can be hypothesised that frontal lesions (precentral gyrus, SLF) may result in an impairment of verbal oral praxis, while temporoparietal lesions may result in an impairment of non-verbal oral praxis.

The involvement of striatocapsular lesions in iOA is in line with previous findings on OA (Tognola and Vignolo 1980; Raade et al. 1991), whereas the role of this region in oral apraxia, and in apraxia in general, remains unclear. The left striatocapsular region may play an active role in oral praxis by communicating with higher cortical regions, as hypothesized in computational models for motor speech production (Eickhoff et al. 2009), and subcortical and cortical lesions have been associated with distinct profiles of impairment in limb apraxia (Hanna-Pladdy et al. 2001). Noticeably, both limb and oral apraxia following subcortical damage mostly do not result from lesions confined to the basal ganglia, but from the additional involvement of peristriatal white matter (e.g. SLF) (Pramstaller and Marsden 1996). In this view, it should be noted that our striatocapsular lesions causing OA reached into the external/extreme capsule. The extreme capsule has been associated with both aphasia and apraxia (see below) and may be responsible for the occurrence of iOA in our patients. Alternatively, diaschisis or cortical hypoperfusion could be involved, as already described for aphasia (Perani et al. 1987; Weiller et al. 1993; Hillis et al. 2004a; Choi et al. 2007).

To further understand the role of the above-mentioned brain regions in verbal and non-verbal oral praxis, we investigated the relation between these symptoms and limb apraxia. At a behavioural level, we observed a significant association between SA + OA and both impairment of pantomime and imitation, whereas the frequency of limb apraxia was not as high as aphasia (approx. 80%). At an anatomical level, the SVR-LSM analysis for SA + OA (Fig. 1c) did not show an association with the typical brain areas involved in limb apraxia. The only exception was the external/extreme capsule, which has been described to play a role in pantomime, based on previous findings from our laboratory (Hoeren et al. 2014; Martin et al. 2016). On the other hand, patient 1 with iOA had a temporoparietal lesion, which included limb apraxia areas: SPL, posterior IPL (angular gyrus), middle temporal gyrus and ATL (Martin et al. 2016; Dressing et al. 2018). Unlike the patients with iSA*,* in this case the typical regions involved in motor speech and language processing were spared. Since this patient manifested with limb apraxia as well (i.e. impairment of imitation; pantomime could not be tested, see Clinical data), and his lesion did not overlap with the subcortical lesions of the other iOA patients, it is not possible to draw a direct link between the occurrence of OA and a specific lesioned area involved in apraxia. Still, it can be argued that iOA relies to some extent on the same anatomical substrates involved in limb apraxia, as already suggested for neurodegenerative patients (Botha et al. 2014).

It has to be pointed out that the tests currently used in a clinical setting for SA and OA inevitably present some intrinsic limitations. More in detail, the diagnosis of SA is based on the identification and weighting of speech behaviour, for example phonematic errors, which alone are not discriminative for SA or aphasia. Also, the subset or combination of features required to diagnose SA is not universally established (Ballard et al. 2016). Internationally, some standardized tools have been developed to diagnose SA, such as rating scales or criteria (Dabul 2000; Feiken and Jonkers 2012; Strand et al. 2014; Ballard et al. 2016), whereas their reliability and clinical applicability still have to be universally confirmed. Furthermore, the tests for both SA and OA intrinsically require, to some extent, semantic skills—namely speech comprehension and pantomime. This may reflect on the clinical association we found between SA/OA and semantic functions, i.e. speech comprehension and pantomime, as well as on the anatomical association with temporal areas. SA + OA were in fact associated with a very small part of the STG (BA22) and patient 1 with iOA showed lesioning of the ATL, which elicits ventral functions in both apraxia (Martin et al. 2016) and aphasia (Schwartz et al. 2009). However, it has to be noted that in the SVR-LSM results within the aphasic group (Fig. 1d), the temporal regions were not significantly associated with SA + OA, suggesting that their involvement rather reflects the co-occurrence of aphasia and not a direct role in verbal and non-verbal oral praxis.

A similar interpretation may be given to the involvement of the external/extreme capsule in SA and OA. Our study did not have sufficient spatial resolution to draw a distinction between extreme and external capsule, but previous evidence showed that the extreme capsule mediates the ventral stream connections for both language processing (i.e. sound to meaning) and pantomime (i.e. observation to meaning), in particular for content errors (Saur et al. 2008; Rijntjes et al. 2012; Hoeren et al. 2014).

Lastly, our results raise the question of how SA and OA can be interpreted, particularly in light of the current models for language and speech production. The role of the insula in language and speech production is still a matter of discussion and this region has been found to relate not only to ventral (sound to meaning), but also to dorsal (sound to articulation) functions (Hickok and Poeppel 2007; Saur et al. 2008; Ackermann and Riecker 2010; Rijntjes et al. 2012). Several functional neuroimaging studies suggest that the insula might be a functional integrational centre between speech and language areas (Cloutman et al. 2012; Oh et al. 2014). One model for motor speech production (Eickhoff et al. 2009), for example, computed a network in which the insula receives information (phonological concepts) from the IFG (BA 44), translates it into vocal motor patterns by communicating with the cerebellum and the basal ganglia, and then converges it into the PMC, from where it is forwarded to the primary motor cortex for speech articulation. Furthermore, insular lesions have been associated with deficits in the control of non-speech oral motor functions, such as OA and dysphagia (Ackermann and Riecker 2010).

Thus, it can be hypothesized that the insula acts as an integrator for oral praxis, between its verbal and non-verbal components, so that its lesioning mostly associates with co-occurring SA and OA. A similar hypothesis was also proposed by Kusch (Kusch et al. 2018), who found that limb-apractic patients with insular lesions had a better prognosis than those with lesions in core lesions of the frontoparietal network. From this point of view, our results suggest that frontal regions (in particular precentral gyrus and SLF) may be exclusively responsible for the verbal component of oral praxis—i.e. SA—while temporoparietal and striatocapsular regions for its non-verbal aspects—i.e. OA.

A different interpretation, under the assumption that verbal and non-verbal oral praxis rely on the same anatomical substrates, may be that a lesion of the insula tends to be associated with a greater impairment of oral praxis in general. Thus, in case of insular damage, both SA and OA may be easily detected with standard clinical testing, resulting in a cohort of patients with mostly co-occurring symptoms. Damage to other regions involved in oral praxis may lead to milder impairments, so that either SA or OA may occasionally be underdiagnosed due to the suboptimal sensitivity of clinical tests (Ballard et al. 2000), resulting in apparent isolated symptoms. Further research is needed to verify these hypotheses.

## Conclusion

The anatomy of verbal and non-verbal oral praxis is likely to consist in a multimodal and highly overlapping network. In our sample of stroke patients, SA and OA mostly co-occur and are predominantly, but not exclusively, associated with the insula. Noticeably, isolated SA and isolated OA occur without insular damage, showing instead frontal lesions (prefrontal gyrus, SLF) or temporoparietal and subcortical lesions, respectively.

These results lend themselves to two possible interpretations. From a task-independent, integrative point of view, it can be postulated that insular lesions are associated with a greater impairment of verbal and non-verbal oral praxis, so that in case of insular damage both SA and OA can be easily detected, while in case of damage of other regions either SA or OA may be underdiagnosed, resulting in apparent isolated symptoms. Alternatively, in a task-dependent point of view, the insula may play an integrative role between verbal and non-verbal oral praxis, so that its lesioning mostly results in co-occurring SA and OA. If the insula is spared, frontal lesions may result in SA, while temporoparietal and subcortical lesions in OA.

The involvement of the above described frontal and temporoparietal areas, which are known to elicit either language- or praxis-related functions, may be influenced by the fact that clinical testing for SA and OA intrinsically and inevitably also test for aphasia and apraxia. Developing refined standardized test in experimental settings, which are able to separate language, speech and praxis components of SA and OA, and do not rely on semantic skills such as speech comprehension and pantomime, is necessary to shed further light on the anatomical relation between SA and OA.

## Declarations

### Availability of data and material (data transparency)

The datasets generated and analysed during the current study are available from the corresponding author on reasonable request.
